# How action influences object perception

**DOI:** 10.3389/fpsyg.2013.00462

**Published:** 2013-07-22

**Authors:** David Chan, Mary A. Peterson, Morgan D. Barense, Jay Pratt

**Affiliations:** ^1^Department of Psychology, University of TorontoToronto, ON, Canada; ^2^Department of Psychology, College of Social and Behavioral Sciences, The University of ArizonaTuscon, AZ, USA

**Keywords:** object perception, magnocellular pathway, action perception, visual pathways, near-hand vision, hand-altered vision

## Abstract

Although object perception is typically associated with the parvocellular (P) pathway, a form of fast “gist” object perception may be due to activity in the magnocellular (M) pathway (Kveraga et al., 2007). Because the M-pathway is typically associated with action, we hypothesized that manipulations of action would influence speeded object perception. In three experiments, participants indicated whether the objects shown in low and high spatial frequency (HSF) images were larger or smaller than a prototypical shoebox. In Experiment 1, participants used a proximal (hands on monitor) or distal (hands on keyboard) hand posture in separate blocks. In Experiment 2, only the proximal hand posture was used, but the hands were either action oriented with palms in (palms toward the stimuli) or non-action oriented with palms out (palms away from the stimuli). In Experiment 3, we used the palms-in proximal hand posture but manipulated the type of visual stimuli such that they were either action oriented (easily grasped) or non-action oriented (not easily grasped). In all three experiments, the advantage in identifying the low spatial frequency (LSF) images was greater when action was primed (proximal hands, palms-in, graspable). Together, these experiments show that the M-pathway is involved in rapid “gist” object perception, and this type of object perception is influenced by action.

## How action influences object perception

One fundamental question regarding visual perception involves what neural streams are responsible for early visual processing. Researchers have discovered that there are two main visual pathways, the Magnocellular pathway (M-pathway) and Parvocellular pathway (P-pathway). Many differences exist between both pathways, with the M-pathway processing information such as motion (Derrington and Lennie, 1984; DeYoe and Van Essen, 1985; Livingstone and Hubel, 1987) and location (Derrington and Lennie, 1984; Chen et al., 2006) and the P-pathway processing information such as color (Derrington and Lennie, 1984) and detailed object features (Maunsell et al., 1990). Thus, broadly speaking, the M-pathway provides visual information related to the planning and production of actions while the P-pathway provides detailed visual information related to the M-pathway (Livingstone and Hubel, 1987; Chen et al., 2006). One noteworthy property of these systems is that they are mutually inhibitory in that when the M-pathway is biased, the P-pathway is inhibited, and vice versa.

Although the P-pathway has traditionally been associated with object perception, Kveraga et al. (2007) have suggested that the M-pathway is also involved in object identification. Their study took advantage of the well-known finding from Wiesel and Hubel (1966) that M-cells are sensitive to low spatial frequency (LSF) information whereas P-cells are sensitive to high spatial frequency (HSF) information. Kveraga et al. presented subjects with line drawings of objects that were either LSF or HSF. The LSF images were all low-luminance and monochromatic, whereas the HSF images were chromatically defined and isoluminant. Subjects were presented with either a LSF or HSF image, and were asked to respond as to whether the object was larger or smaller than a typical shoebox. Results showed a benefit for LSF images. Reaction times (RTs) for LSF images were on average 105 ms faster than HSF images and the overall accuracy of the LSF images were significantly better than the HSF images. Kveraga et al. hypothesized that these findings were due to LSF “gist” information being rapidly carried by the M-pathway to support rapid object perception. This gist image activates predictions about candidate objects similar to the image in their LSF appearance, which are in turn fed back from the frontal lobe to ventral object recognition regions to facilitate distinction among these object candidates.

If the M-pathway is involved in rapid object identification, it follows that processes also supported by the M-pathway might exert some influence on object perception. Specifically, as the M-pathway is thought to underlie the action systems in primates (Wiesel and Hubel, 1966), we examined the role of action on object perception. To accomplish this, we made use of a manipulation of hand posture first reported by Abrams et al. (2008). Across three experiments, Abrams et al. had participants assume either a proximal hand posture (where both hands were up toward the computer screen) or a distal hand posture (where both hands were down toward the keyboard). They found that the proximal hand posture resulted in steeper search slopes, greater inhibition of return (IOR), and increased attentional blinks compared to the distal hand posture. Using the same manipulation, Davoli et al. (2010) presented participants with sensible and non-sensical sentences and found that semantic processing was impoverished near the hands. They also presented participants with a traditional Stroop inference task and found that the magnitude of the effect was dramatically reduced when subjects adopted a proximal hand posture. In addition, Tseng and Bridgeman (2011) found improved change detection performance with hands in proximal position. Thus, hand posture has been found to have a robust effect of a variety of tasks.

In order to explain the constellation of effects resulting from placing both hands on the computer monitor where the stimuli are being presented, Gozli et al. (2012) proposed that a proximal hand posture biased processing in the M-pathway and a distal hand posture biased processing in the P-pathway. In their study, participants completed a spatial gap task in which they estimated varying gap sizes and a temporal gap task in which they estimated varying stimulus onset asynchronies (SOAs). In both conditions, participants assumed either a proximal (hands close to the stimuli, thus priming action) or distal (hands further from the stimuli and not priming action) hand posture. Gozli et al. reported that when participants had a proximal hand posture, they were better at the temporal gap task, as predicted by the higher temporal resolution associated with the M-pathway. When participants assumed a distal hand posture, they were more accurate at the spatial gap task, as predicted by the higher spatial resolution associated with the P-pathway. Thus, this experiment provides the initial evidence different hand postures biases activity in the two visual systems.

Further support for the notion that a proximal hand posture biases M-pathway processing comes from Goodhew et al. (2013) using object substitution masking (OSM). An OSM task involves a sparse (e.g., four dot) temporally-trailing mask obscuring the visibility of a briefly-presented target (c.f. recent review on OSM, Goodhew et al., in press). Treating OSM as a problem of temporally segregating the mask from the target, Goodhew et al. reasoned that if a proximal hand posture biases M-pathway activity and improves temporal resolution, it should reduce the effect of the masks. Across two experiments, this is indeed what they found, providing more evidence that a proximal hand posture biases the M-pathway while a distal hand posture biases the P-pathway. Taken together, the Gozli et al. (2012) and Goodhew et al. (2013, in press) studies indicate that manipulating hand posture is a useful tool to investigate whether actions can influence object perception.

To address the question of whether objects are affected by action, a similar object perception task to that of Kveraga et al. (2007) was used in the three experiments of this study. That is, participants were shown either LSF or HSF images of objects and asked to indicate (either with a keypress or a mouse click) whether the object was larger or smaller than a prototypical shoebox. Different action manipulations, however, were used across the three experiments. The first experiment used the same hand posture manipulation as Abrams et al. (2008) and Gozli et al. (2012); both hands either proximal or distal. Experiment 2 used two variations of the proximal hand posture; palms-in (toward the display) or palms-out (away from the display). The final experiment used a single palms-in proximal hand posture but the type of visual image was manipulated (objects that were either easy or difficult to act on). These three interrelated action manipulations were used to test the idea that the action-based M-pathway is involved in rapid object perception using gist processing.

We hypothesize that anything which primes “action” will in turn bias the M-pathway. According to the hypothesis proposed by Kveraga et al. (2007), the M-pathway is involved in some form of fast object perception, in that the M-pathway quickly uses LSF information to quickly provide a blurred template of an object, which is then filled in by our top-down memories of objects. They coined this “gist” processing, as they argued that the M-pathway just used the “gist” of the information available from the object for speeded object perception. To test this hypothesis, we exploited the fact that the M-pathway is also an action pathway, responsible for processing motion and action. We therefore surmised that priming action would also prime the M-pathway. This priming biases the M-pathway, which in turn biases the processing of “gist” information, and in our case, LSF information.

## Experiment 1

In the first experiment participants either assumed a proximal (hands up) or distal (hands down) hand posture and were instructed to determine if the LSF and HSF images represented objects that were larger or smaller than a prototypical shoebox. Although the task is a relative size judgment task, the task requires participants to correctly identify the object before making an accurate assessment of the size, since the actual stimuli provides little information about the actual size of the object. Therefore, if the proximal hand posture biases M-pathway processing, and rapid gist object perception relies on the M-pathway, we predict that the LSF advantage reported by Kveraga et al. (2007) will be greater with the proximal hand posture than the distal hand posture.

### Subjects and apparatus

Twelve University of Toronto undergraduates (mean age = 22.8; 8 females) participated in Experiment 1. Subjects received course credit for their participation. All subjects reported normal or corrected-to-normal vision and none were aware of the hypothesis tested. The experiment was conducted in a dimly lit, sound-attenuated testing room. Visual stimuli were presented on a CRT computer monitor with a refresh rate of 85 Hz. A chin and head rest maintained a viewing distance of 48 cm. Responses were collected on a standard keyboard with a key press (“F” or “J” key).

### Procedure and design

**Figure 1:**
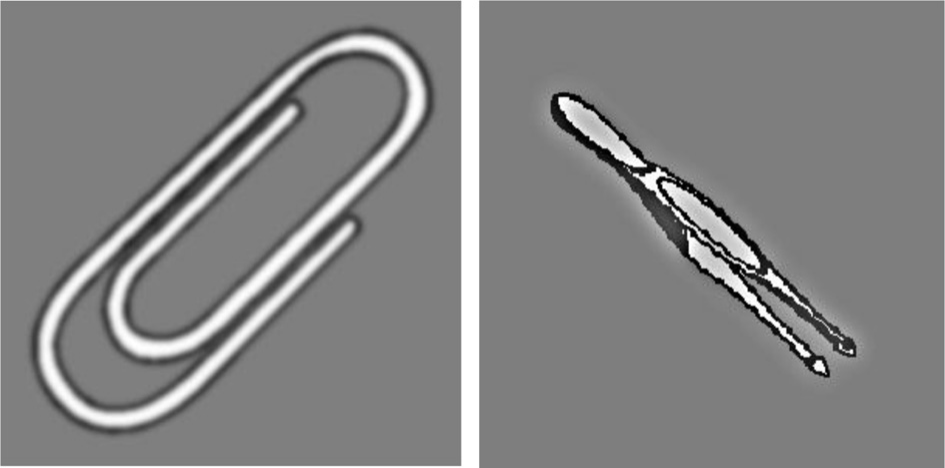
**Left:** LSF image. **Right:** HSF image.

**Figure 2:**
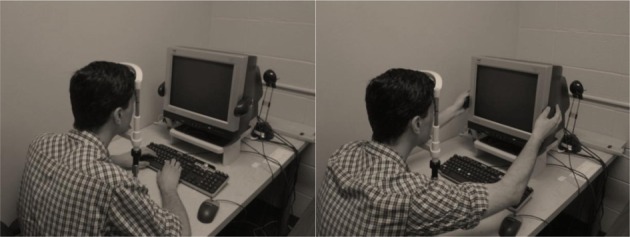
**Left:** Distal hand position. **Right**: Proximal hand position.

All stimuli were presented on a gray background. Each trial began with a white fixation cross in the middle of screen for between 250 and 750 ms, to prevent participants from anticipating the onset of the stimulus. Next, a grayscale object (5° × 5°) replaced the fixation cross at the center of the screen. The object was either HSF or LSF created by either a high pass filter, or a Gaussian blur of 5° (σ = 2.5, 0.027°/cycle), respectively (Figure 1). The spatial frequency of the image was randomized across every trial, and each image was shown as both LSF and HSF. Participants were instructed to make a size judgment response by indicating whether the object was larger (“J” key) or smaller (“F” key) than a shoebox. Participants completed this task both with their hands proximal (hands on the computer monitor) or distal (hands on the keyboard) to the stimuli in counterbalanced blocks of 320 trials each (Figure 2). Thus, there were four conditions in total (HSF/hands proximal, HSF/hands distal, LSF/hands proximal, and LSF/hands distal). There were a total of 80 unique images taken from the International Picture-Naming Project (Szekely et al., 2003), each of which was viewed four times across the four conditions. The stimulus set consisted of everyday items (e.g., lamps, backpacks, saltshaker) that were matched for name familiarity, in that previous studies showed no advantage for image naming across all the stimuli (Szekely et al., 2003).

### Results and discussion

**Figure 3:**
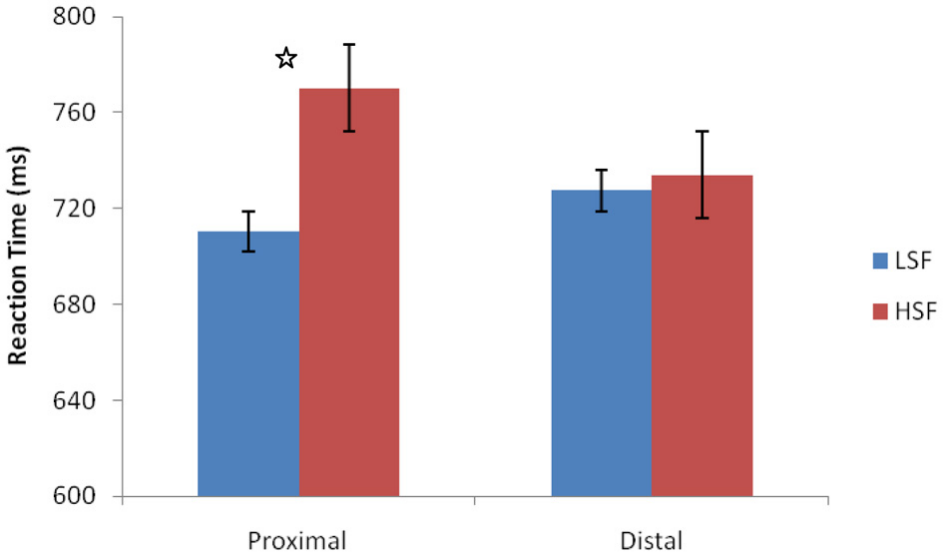
**Reaction times for proximal and distal hand postures.** No main effect of hand posture or spatial frequency. However, significant interaction between spatial frequency and hand posture. Error bars represent standard errors of means. ^*^ *p* < 0.05.

Trials with RTs less than 100 ms or greater than 2 standard deviations from the participants mean RT were removed prior to analysis (less than 2% of all trials). The RTs were analyzed with a repeated measures ANOVA with within-subjects factors of hand posture (proximal or distal) and spatial frequency (LSF or HSF) (Figure 3). No main effect of spatial frequency [*F*_(1, 11)_ = 4.262, *p* = 0.063, η^2^_*p*_ = 0.279] or hand posture [*F*_(1, 11)_ = 0.063, *p* = 0.807, η^2^_*p*_ = 0.006] was found. However, a significant interaction between hand posture and spatial frequency indicated that the LSF advantage was larger when hand posture was proximal rather than distal, [*F*_(1, 11)_ = 6.377, *p* = 0.028, l, η^2^_*p*_ = 0.367]. A *post-hoc t*-test demonstrated that when hands were proximal to the stimuli, participants were faster at processing LSF than HSF information [*t*_(11)_ = 2.418, *p* = 0.034, *d* = 1.459], but not when the hands were distal [*t*_(11)_ = 0.581, *p* = 0.573, *d* = 0.350]. The finding that hand posture affected rapid object perception for LSF images supports Kveraga et al.'s (2007) hypothesis that gist processing is performed by the M-pathway.

One result worth noting is that, compared to the distal condition, there seemed to be an increase in RTs for HSF objects in the proximal condition vs. the distal condition, instead of a decrease in RTs for LSF objects. This is most likely due to the mutually inhibitory relationship between the M and P-pathways (Yeshurun and Levy, 2003), as a bias toward the M-pathway also means an inhibitory effect toward the P-pathway, which would tend to increase RT in the distal condition.

## Experiment 2

Although the results from Experiment 1 are consistent with the notion that rapid gist object perception is supported by the M-pathway, an alternative interpretation needs to be considered. Specifically, it may be that by having the hands proximal to the computer screen, people are attending to the stimuli differently because the images are in peripersonal space. In other words, the effect we have found may be due to a change in attention with hand posture, not a bias in action-based M-pathway processing. Indeed, there is evidence to support this alternative. For example, Reed et al. (2006) presented participants with a standard covert attention task, where on each trial, a highly predictive visual cue (70%) indicated the probability of the target appearing at that location. Participants responded when a target appeared at either the cued location (validly cued trial) or the uncued location (invalidly cued trial). Furthermore, participants had their hands placed beside one of the target locations (either to the left or right of the computer screen). Reed et al. reported that participants were faster at responding to valid cues at the locations closer to their hands, suggesting that attention was biased toward hand locations. Therefore, it may be that the findings of our first experiment were driven by increased attention near hand space in the proximal hand condition.

In order to test between the action-based and attention-based possibilities, a second experiment was conducted which only the proximal hand posture was used. In this case, however, we manipulated the direction of the palms, such that the palms were facing toward (palms-in) or away from (palms-out) the object images (Figure 3). If the effects found in the first experiment were due to activation of the action-based M-pathway, we would expect to see a greater LSF advantage with palms-in (because the object images are in action space) than with palms-out (because the objects images are out of action space). Our rationale for this is that a palms-in posture will provide a stronger bias of the M-pathway because we typically act on objects with our palms rather than the backs of our hands. If, however, the effects were due to greater attention to the images in the proximal condition, there should be no differences in the LSF advantage between the two condition because the hands are the same distance from the stimuli.

### Subjects and apparatus

Fourteen University of Toronto undergraduates (mean age = 18.1; 11 females) participated in Experiment 2. Based on the effect size from the critical interaction in Experiment 1, 14 subjects would achieve 88% power. Subjects received course credit for their participation. All subjects reported normal or corrected-to-normal vision and none were aware of the hypothesis tested. The experiment was conducted in a dimly lit, sound-attenuated testing room. Visual stimuli were presented on a CRT computer monitor with a refresh rate of 85 Hz. A chin and head rest maintained a viewing distance of 48 cm.

### Procedure and design

**Figure 4:**
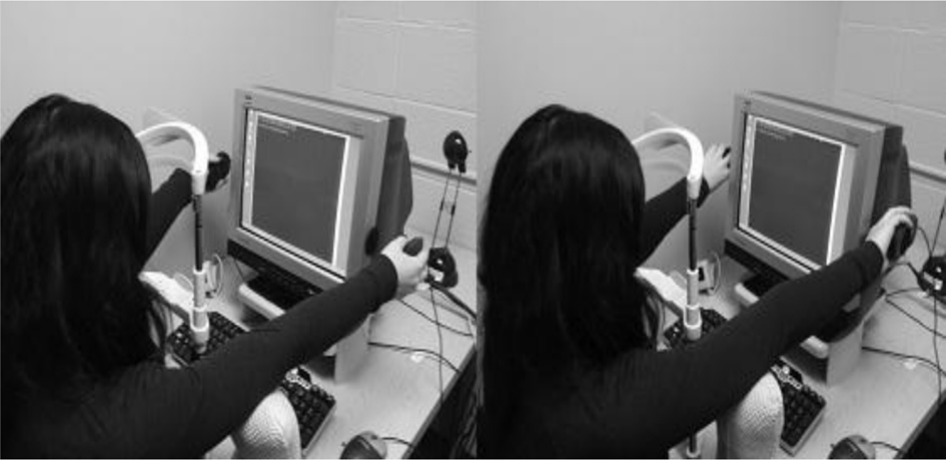
**Left:** Palms-in hand posture. **Right:** Palms-out hand posture.

The design of the second experiment was the same as Experiment 1 except that two proximal hand postures are used. One hand posture is the palms-in posture, which is identical to the proximal hand posture of Experiment 1. The second hand posture is the palms-out posture, in which participants pointed their palms out away from the computer screen (Figure 4). In both these postures, the distance between the stimuli and their hands remained constant. In order to help facilitate comfort in the palms-out posture, pillows were used to brace the arms and elbows, and were also used in the palms-in posture to keep consistent between conditions.

### Results and discussion

**Figure 5:**
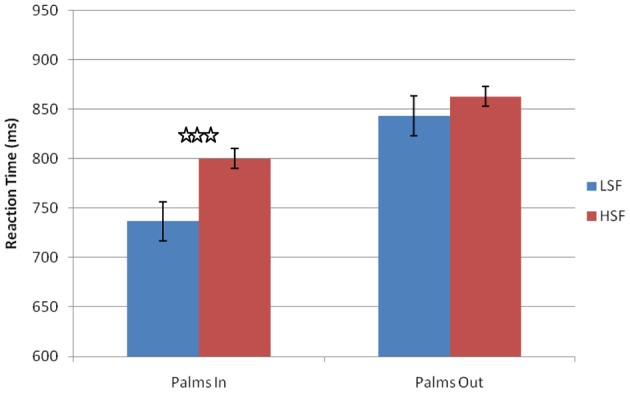
**Results from Experiment 2.** Main effect of spatial frequency and significant interaction between spatial frequency and hand posture. Error bars represent standard error of means. ^***^p < 0.001.

Trials with RTs less than 100 ms or greater than 2 standard deviations from the participants average RT were removed prior to analysis (less than 3% of all trials). The RTs were analyzed with a repeated measures ANOVA with within-subjects factors of hand posture (palms-in or palms-out) and spatial frequency (LSF or HSF). There was a main effect of spatial frequency [*F*_(1, 12)_ = 12.543, *p* = 0.004, η^2^_*p*_ = 0.491], with faster RTs for LSF images (Figure 5). A main effect of palms was found [*F*_(1, 12)_ = 8.920, *p* = 0.011, η^2^_*p*_ = 0.663], with faster responses with palms-in than palms-out. This may have been due to the fact that having palms-in was generally more comfortable for the participants. Importantly, the interaction between palm position and spatial frequency was significant [*F*_(1, 12)_ = 25.572, *p* = 0.0001, η^2^_*p*_ = 0.663]. A *post-hoc t*-test confirmed that participants had an advantage at identifying LSF images when their palms were in, [*t*_(13)_ = 5.346, *p* = 0.001, *d* = 2.965], but not when their palms were out [*t*_(13)_ = 1.454, *p* = 0.17, *d* = 0.806]. This interaction, driven by the shorter RTs for the LSF images in the palms-in hand posture, provides strong evidence that priming action does indeed influence object perception. Thus, these data provide additional support to the idea that priming action biases the M-pathway, which allows our visual system to prioritize LSF information.

## Experiment 3

The findings of the first two experiments converge to suggest that action, as manipulated through hand position, can alter object perception via the M-pathway. In our last experiment, our aim was to confirm this interpretation by using a paradigm that required only a single hand position. In Experiment 2, it may be the case that because of the nature of the palm orientation, there are still attentional effects at play. Indeed, Reed et al. (2010) proposed that a larger number of bimodal neurons represent our palms than the back of our hands, which allows for greater attention near the palms. Therefore, in the present experiment, we used a single proximal hand posture but this time manipulated action through the stimuli being presented. It can be reasoned that if priming action induces a bias toward the M-pathway, then stimuli that are “action oriented” should yield similar results to the first two experiments (i.e., faster responses for LSF action-oriented images). In our world, there are many objects that are more easily associated with action, such as objects that are graspable or easily manipulated with our hands. It would stand to reason that these objects would produce an effect of action, as opposed to objects that are harder to manipulate with our hands. Therefore, our third experiment used objects that were either easily graspable or easily manipulated with the hands (action-oriented) or objects that were not (non-action oriented) in order to bias the M-pathway without changing hand posture (Figure 5).

### Subjects and apparatus

Eight University of Toronto undergraduates (mean age = 19.75; 2 females) participated in Experiment 3. Based on the mean effect size of the critical interaction in Experiments 1 and 2, this achieved 82% power. Subjects received course credit for their participation. All subjects reported normal or corrected-to-normal vision and none were aware of the hypothesis tested. The experiment was conducted in a dimly lit, sound-attenuated testing room. Visual stimuli were presented on a CRT computer monitor with a refresh rate of 85 Hz. A chin and head rest maintained a viewing distance of 48 cm.

### Procedure and design

**Figure 6:**
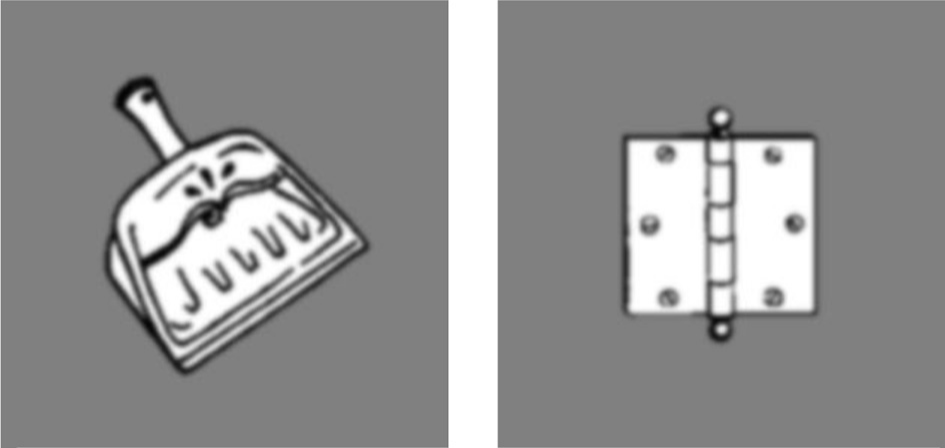
**Left:** Example of LSF action-oriented object. **Right**: Example of LSF non-action-oriented object.

The design of the third experiment was the same as that of Experiment 1 except for two differences. First, only the proximal hand posture (with palms facing in) was used. Second, the stimuli were changed to match two categories: action-oriented objects and non-action oriented objects. Action oriented objects were objects such as tools that met two criteria. The first was that the object had to elicit a natural action that is performed by the hands. The second is that the action done by the hands had to accomplish a goal or a task. For example, a dustpan is an object that elicits an action from the hands (grasping it and moving it), while doing so accomplishes a goal (picking up dust) (Figure 6). Non-action oriented objects were objects that did not meet these 2 requirements (e.g., a door hinge as in Figure 6). Forty unique objects were used in Experiment 3 and all objects were matched for image size. All objects were taken from the same naming database as Experiment 1. The task and responses also remained the same as Experiment 1. Experiment 3 consisted of 160 trials with action and non-action oriented objects randomly intermixed, with each item being presented as both LSF and HSF.

### Results and discussion

**Figure 7:**
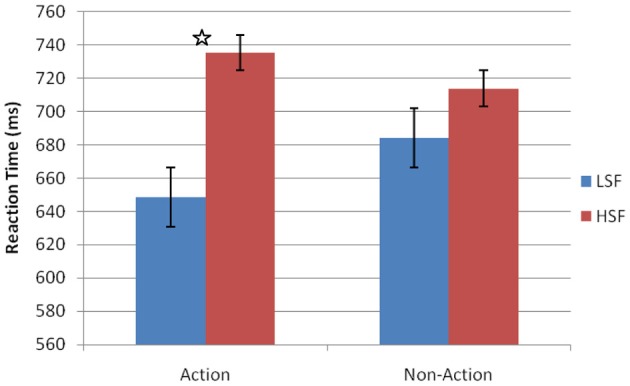
**Results for Experiment 3.** Main effect of spatial frequency and significant interaction between stimulus type and spatial frequency. Error bars represent standard error of means. ^*^p < 0.05.

Trials with RTs less than 100 ms or greater than 2 standard deviations from the participants average RT were removed prior to analysis (less than 1% of all trials). The RTs were analyzed with a repeated measures ANOVA with within-subject factors of stimulus type (action or non-action) and spatial frequency (LSF or HSF) (Figure 7). There was no main effect of object type [*F*_(1, 7)_ = 3.134, *p* = 0.122, η^2^_*p*_ = 0.139]. But we found a main effect of spatial frequency [*F*_(1, 7)_ = 17.866, *p* = 0.004, η^2^_*p*_ = 0.718], indicating that LSF images were processed faster. Furthermore, we found a significant interaction between object type and spatial frequency [*F*_(1, 7)_ = 9.560, *p* < 0.018, η^2^_*p*_ = 0.577], driven by faster RTs to LSF than HSF images when they were “action-oriented” [*t*_(7)_ = 5.508, *p* = 0.001, *d* = 4.164], not when they were “non-action oriented” [*t*_(7)_ = 2.478, *p* = 0.071, *d* = 1.873]. (The marginal difference between the LSF and HSF conditions with non-action oriented objects may have been driven by the palms in posture that was assumed in Experiment 3, which we know from Experiment 2 biases the M-pathway.) Again, these data suggest that participants were faster at processing LSF information when action was primed through the stimulus type. By holding the hand posture constant, we were able to demonstrate that action priming still biases the M-pathway, which allows the visual system to prioritize LSF information.

## General discussion

The main question that we addressed in these experiments was whether or not action influences object perception. This question arose from Kveraga et al.'s (2007) hypothesis that rapid object perception in accomplished by the M-pathway. In three experiments, we manipulated action in different ways to provide a clear answer: object perception is affected by hand posture, an action-based manipulation that influences M-pathway processing. The first two experiments showed that the LSF advantage (an index of M-pathway processing) was greater with the proximal hand posture (Experiment 1) and with the palms-in posture (Experiment 2), hand postures that prime action and are therefore expected to prime the M-pathway. Experiment 3 showed that the type of images—whether they were action-oriented or not—could also facilitated the LSF advantage, while hand posture was held constant. Thus, the present results show that priming the M-pathway by priming action can indeed influence object perception.

This study provides support for the hypothesis proposed by Kveraga et al. (2007) that the M-pathway is involved in some form of object perception. It seems likely that this M-pathway involvement is in rapid object perception, which allows the visual system to make quick decisions based on limited LSF information, which is a capability that serves an evolutionary benefit. The M-pathway receives the majority of its input from the rod cells in the retina, which make up most of our field of view. Being able to quickly extract sufficient information about objects in the periphery (predators and prey, edible and non-edible, for example) would be a useful aid in survival. Indeed, when perceiving whether objects may harm or can be manipulated in useful actions, it is not completely necessary to process fine spatial detail, but rather the gist of the object shape allows us to determine its relevance to us. Therefore, priming the M-pathway facilitates speeded object recognitions that allow fast and informed decisions – capacities that would have conferred survival benefits.

Beyond implications for object perception, this research also provides insight into the hand posture literature. The present experiments offers further evidence that the M-pathway drives the effects observed when proximal hand postures are adopted. The increased temporal resolution and the biases toward specific types of information are consistent with facilitation of the M-pathway through the use of action. For example, Gozli et al. (2012) found superior temporal resolution, a characteristic of M-pathway processing, when participants assumed a proximal hand posture. This again could be seen as an evolutionary tool, as the ability to process action-oriented information at an increased speed would have considerable survival value.

Finally, one implication of this line of research is that there might not be a “neutral posture” from which to examine various aspects of perception or cognition. Since the inception of Cognitive Psychology, experiments have been conducted in a sitting posture with participants' hands proximal to the device they must use to make responses (e.g., keyboard, response box, pen and paper). The tacit assumption has been that this posture would produce effects similar to any other posture, or at least be neutral in terms of any specific influences on the task at hand. In fact, this posture appears to bias P-pathway processing, thus potentially altering performance. Thus, it may be useful to re-examine perceptual and cognitive studies that involve potential trade-offs between the M- and P-pathways. However, one thing to note is that in our experiments, we were able to replicate the LSF advantage reported by Kveraga et al. (2007) only when we primed the M-pathway, either by palm positions (Experiments 1 and 2) or by the use of action-oriented objects combined with a favorable palm position (Experiment 3). Perhaps it is necessary to strongly bias the M-pathway processing in order to see the LSF vs. HSF differences. Consistent with this possibility, Kveraga et al. (2007) combined the presence vs. absence of luminance differences with LSF and HSF images. Since we know that the luminance also drives the M-pathway, it might be the case that only under strong biases do we see this fast processing of the M-pathway. Therefore, this observation raises a question regarding the extent to which fast processing in the M-pathway facilitates perception under normal conditions, and whether only under strong biases does the M-pathway provide these observational advantages for LSF information.

### Conflict of interest statement

The authors declare that the research was conducted in the absence of any commercial or financial relationships that could be construed as a potential conflict of interest.
